# In vivo imaging of sterile microglial activation in rat brain after disrupting the blood-brain barrier with pulsed focused ultrasound: [18F]DPA-714 PET study

**DOI:** 10.1186/s12974-019-1543-z

**Published:** 2019-07-25

**Authors:** Sanhita Sinharay, Tsang-Wei Tu, Zsofia I. Kovacs, William Schreiber-Stainthorp, Maggie Sundby, Xiang Zhang, Georgios Z. Papadakis, William C. Reid, Joseph A. Frank, Dima A. Hammoud

**Affiliations:** 1 Hammoud Laboratory, Center for Infectious Disease Imaging, Clinical Center, National Institutes of Health, 10 Center Drive, Building 10, Room 1C-368, Bethesda, MD 20892 USA; 2 Frank Laboratory, Radiology and Imaging Sciences, Clinical Center, National Institutes of Health, Bethesda, MD USA; 3 Center for Neuroscience and Regenerative Medicine, Uniformed Services University of the Health Sciences, Bethesda, MD USA; 4 Imaging Probe Development Center, National Heart, Lung, and Blood Institute, National Institutes of Health, Rockville, MD USA; 5 National Institute of Biomedical Imaging and Bioengineering, National Institutes of Health, Bethesda, MD USA; 6 University of Texas, MD Anderson Cancer Center, Houston, USA; 7 Department of Radiology, Howard University, Washington DC, USA; 8 Institute for Biomedical Engineering, Swiss Federal Institute of Technology, Zurich, Switzerland; 9 Department of Radiology, University of Crete and Department of Medical Imaging Heraklion University Hospital, Crete, Greece

**Keywords:** Neuroinflammation, [18F]DPA-714 PET, Pulsed focused ultrasound, Magnetic resonance imaging, Translocator protein

## Abstract

**Background:**

Magnetic resonance imaging (MRI)-guided pulsed focused ultrasound combined with the infusion of microbubbles (pFUS+MB) induces transient blood-brain barrier opening (BBBO) in targeted regions. pFUS+MB, through the facilitation of neurotherapeutics’ delivery, has been advocated as an adjuvant treatment for neurodegenerative diseases and malignancies. Sterile neuroinflammation has been recently described following pFUS+MB BBBO. In this study, we used PET imaging with [18F]-DPA714, a biomarker of translocator protein (TSPO), to assess for neuroinflammatory changes following single and multiple pFUS+MB sessions.

**Methods:**

Three groups of Sprague-Dawley female rats received MRI-guided pFUS+MB (Optison™; 5–8 × 10^7^ MB/rat) treatments to the left frontal cortex and right hippocampus. Group A rats were sonicated once. Group B rats were sonicated twice and group C rats were sonicated six times on weekly basis. Passive cavitation detection feedback (PCD) controlled the peak negative pressure during sonication. We performed T1-weighted scans immediately after sonication to assess efficiency of BBBO and T2*-weighted scans to evaluate for hypointense voxels. [18F]DPA-714 PET/CT scans were acquired after the BBB had closed, 24 h after sonication in group A and within an average of 10 days from the last sonication in groups B and C. Ratios of T1 enhancement, T2* values, and [18F]DPA-714 percent injected dose/cc (%ID/cc) values in the targeted areas to the contralateral brain were calculated. Histological assessment for microglial activation/astrocytosis was performed.

**Results:**

In all groups, [18F]DPA-714 binding was increased at the sonicated compared to non-sonicated brain (%ID/cc ratios > 1). Immunohistopathology showed increased staining for microglial and astrocytic markers in the sonicated frontal cortex compared to contralateral brain and to a lesser extent in the sonicated hippocampus. Using MRI, we documented BBB disruption immediately after sonication with resolution of BBBO 24 h later. We found more T2* hypointense voxels with increasing number of sonications. In a longitudinal group of animals imaged after two and after six sonications, there was no cumulative increase of neuroinflammation on PET.

**Conclusion:**

Using [18F]DPA-714 PET, we documented in vivo neuroinflammatory changes in association with pFUS+MB. Our protocol (utilizing PCD feedback to minimize damage) resulted in neuroinflammation visualized 24 h post one sonication. Our findings were supported by immunohistochemistry showing microglial activation and astrocytosis. Experimental sonication parameters intended for BBB disruption should be evaluated for neuroinflammatory sequelae prior to implementation in clinical trials.

**Electronic supplementary material:**

The online version of this article (10.1186/s12974-019-1543-z) contains supplementary material, which is available to authorized users.

## Introduction

The highly selective nature of the blood-brain barrier (BBB) limits the effective delivery of neurotherapeutics to the brain. The BBB consists of a combination of specialized endothelial cells, junctional complexes between adjacent endothelial cells, basement membranes, and perivascular structures comprising the neurovascular unit (NVU) [1, 2]. Several strategies have so far been employed to effectively bypass the BBB for enhanced localized delivery of drugs, genes, antibodies, and nanoparticles [3–7]. Among these techniques, noninvasive MRI-guided pulsed focused ultrasound with intravenous injection of microbubbles (pFUS+MB) has emerged as a promising approach for targeted transient BBB opening (BBBO) [8–12].

Although the exact mechanism of BBBO is still unclear, it has been postulated that pFUS+MB generates acoustic radiation forces in association with the intravascular MB which results in oscillations leading to the propagation of pressure waves and interaction with and through the NVU endothelium, resulting in decreased tight junction integrity [13, 14]. This process has long been assumed to be benign in nature with minimal damage. More recently, however, a rapid increase in damage-associated molecular pattern (DAMP) factors [15–17] was observed following pFUS+MB, with increased expression of heat shock protein 70 (HSP70) and proinflammatory cytokines including tumor necrosis factor alpha (TNFα), interleukin (IL)1α, IL1β, IL18, and interferon gamma (IFNγ) in sonicated animal brains [14, 18], suggesting a neuroinflammatory process.

MR imaging with gadolinium chelate contrast administration has been used extensively in the evaluation of pFUS+MB BBBO in animal models. The efficacy of BBBO post pFUS+MB is generally demonstrated on contrast-enhanced T1-weighted images in animal models and clinically as areas of contrast extravasation [12, 19–22]. The MRI changes detected following sonication include T2 hyperintensities, microhemorrhages, and enlarged ventricles [14, 23, 24]. However, MRI cannot directly visualize inflammatory changes associated with pFUS+MB treatments. On the other hand, positron emission tomography (PET) imaging can assess neuroinflammation, non-invasively, through targeting of the translocator protein (TSPO), an outer mitochondrial membrane receptor known to be upregulated in activated microglia and macrophages [25, 26]. One TSPO ligand, ^18^F-DPA714, has already shown promise in detecting microglial activation in preclinical disease models [27–29] as well as in patients with Alzheimer’s disease (AD) and stroke [30, 31].

In this study, we used [18F]DPA-714 as an in vivo biomarker of neuroinflammation in pFUS+MB-treated rats. First, we probed for effective BBBO immediately after sonication with MRI and assessed for resolution of BBBO 24 h after sonication. We compared [18F]DPA-714 binding in the sonicated left frontal cortical and right hippocampal regions to the contralateral intact brain after one, two, and six sonications. We evaluated the potential cumulative effect of multiple sonications on the inflammatory response by longitudinally evaluating a subgroup of rats after receiving two and six weekly pFUS+MB treatments. Finally, we confirmed our PET findings with immunofluorescent staining for microglial activation and astrocytosis.

## Methods

### Animals

All experiments were approved by the Animal Care and Use Committee (ACUC) of the Clinical Center (CC) at the National Institutes of Health (NIH). Sprague-Dawley female rats (8–10 week old, *n* = 21) were used for all experiments (Charles River Laboratory, Wilmington, MA). Animals were housed in the small animal housing facility at an ambient temperature of 72 ± 2 °F (21–23 °C) which is within the range suggested by the national research Council [32]. Animals were allowed free access to food and water, with a 12 h light/dark cycle. All MR and PET imaging experiments were performed within the light cycle. The animals were kept warm during various procedures using heating pads.

The animals were divided into three groups. Group A rats (*n* = 6) received one pFUS+MB treatment and were PET imaged 24 h after sonication. Group B rats (*n* = 5) received two weekly sonications (2×) and underwent PET 13–14 days after the second sonication. Finally, group C rats (*n* = 5) received six weekly sonications (6×) and were imaged 7–9 days after the last sonication session.

**Fig. 1:**
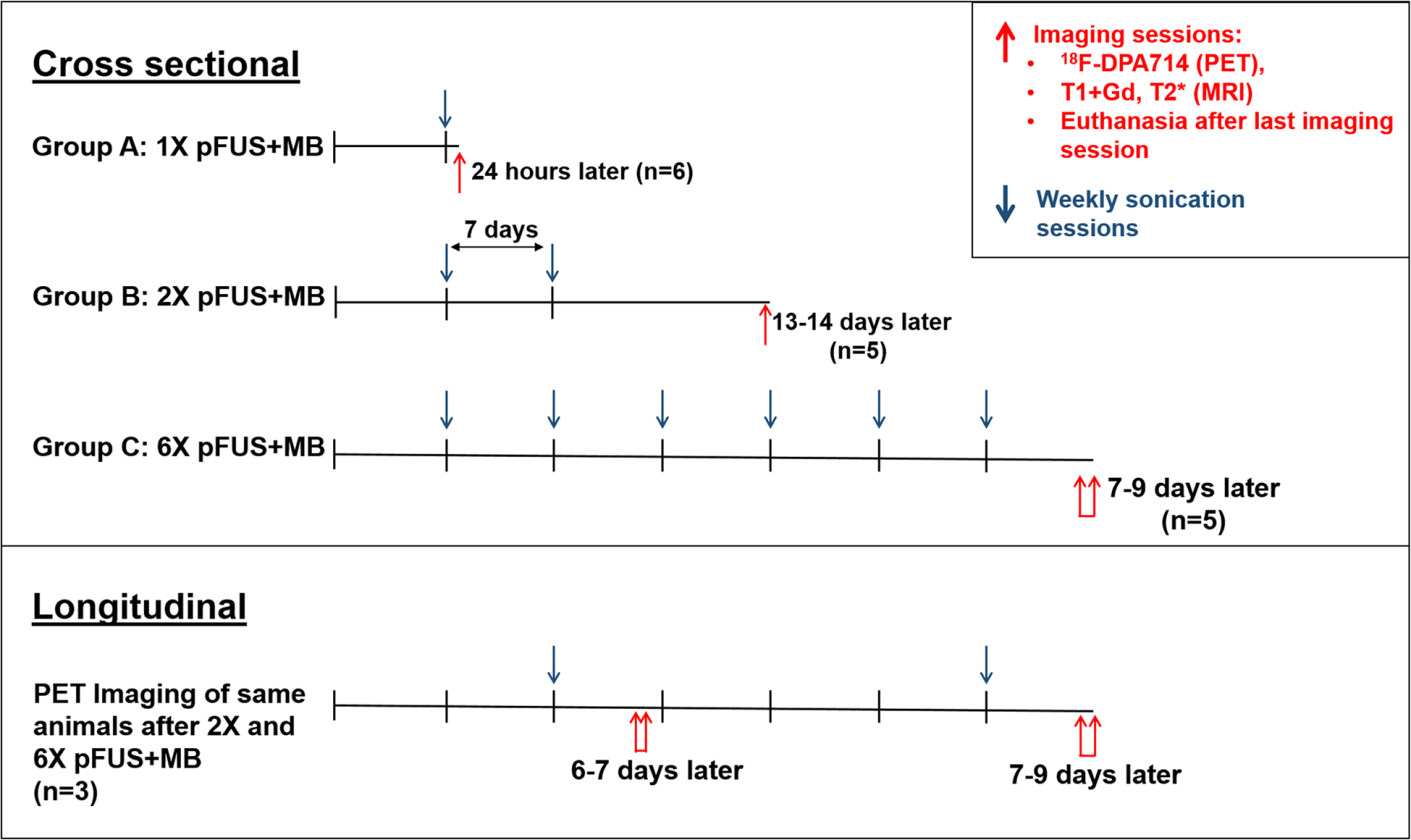
Schematic representation of the pFUS+MB, PET, and MR imaging sessions for cross-sectional and longitudinal components

Three animals from group C (six sonications) were also imaged after two sonications and as such were used for longitudinal imaging (*n* = 3, PET imaging performed 5–6 days after the second sonication and 7–9 days after the sixth sonication). The imaging data from the second sonication in the longitudinal group was not used for the cross-sectional analysis. All animals were euthanized following their last PET imaging session (Fig. 1).

In a separate experiment to document reversal of BBBO after sonication, we performed MR imaging on five rats immediately after sonication as well as 24 h later, using pre and post contrast T1-weighted images.

### MRI-guided pFUS+MB and MR imaging (in vivo and ex vivo)

Rats were first anesthetized with isoflurane (1–3.5%) on 100% O_2_ as previously described [14, 24]. To determine the pFUS targeting coordinates in the left frontal cortex and right hippocampal regions, axial turbo-spin echo (TSE) T2-weighted images with repetition time/echo time (TR/TE = 2000/60 ms) of the rat brain were acquired on a 3T MRI scanner (Achieva, Philips Healthcare, Andover, MA) using a surface coil (RK-100 or LP-100; FUS instruments, Toronto, ON). At the time of the final PET session, group A animals weighed 171.3 ± 10.1 g, group B animals weighed 243.4 ± 12.9 g, and group C animals weighed 276.9 ± 20.8 g. Before each pFUS+MB session, each rat was infused with 100 μL gadopentetate dimeglumine (Gd-DTPA, Magnevist®, Bayer Healthcare Pharmaceuticals, Inc., MA) via tail vein. Thirty seconds prior to initiating pFUS, an intravenous infusion of 100 μL Optison™ (GE Healthcare, Little Chalfont, Buckinghamshire, UK) (range 584–361 μL/kg, at a rate of 1.66 μL/s) was performed over 30 s as previously described [24, 33]. Each animal in this study received the same number of Optison™ MB (5–8 × 10^7^) with an intravascular half-life of 0.72 min because animals were receiving 100% oxygen [24, 33]. Sonication was targeted to the left frontal and right hippocampal regions with non-overlapping 2-mm-diameter focal regions and with a time lapse of 5 min between the two targeted regions. pFUS was performed using passive cavitation detection (PCD) in which the peak negative pressure (PNP) was changed in real time using proprietary hardware and software from the manufacturer (FUS Instruments, Toronto, ON) while monitoring the ultraharmonic frequencies at 1.5*f*_*0*_ and 2.5*f*_*0*_ to correct US pressures if the frequency exceeded 3.5 compared to fast Fourier transform (FFT) baseline [34]. The algorithm for PCD feedback included 10 US bursts at 0 W input power (0 MPa) that were used as a baseline. The starting PNP for all groups was 0.144 MPa with incremental increases of 0.008 MPa with each pulse repetition frequency (PRF = 0.5–0.6 Hz). The pFUS parameters were as follows: transducer center frequency = 548 kHz (FUS Instruments), focal diameter = 0.8, active diameter = 7.5 cm, burst = 10 ms, and duty cycle < 1% with planned 100 sonications per focal spot over 120 s. Axial T1-weighted images were acquired immediately after sonication to document the location and extent of gadolinium extravasation (TR/TE = 350/12 ms).

**Fig. 2:**
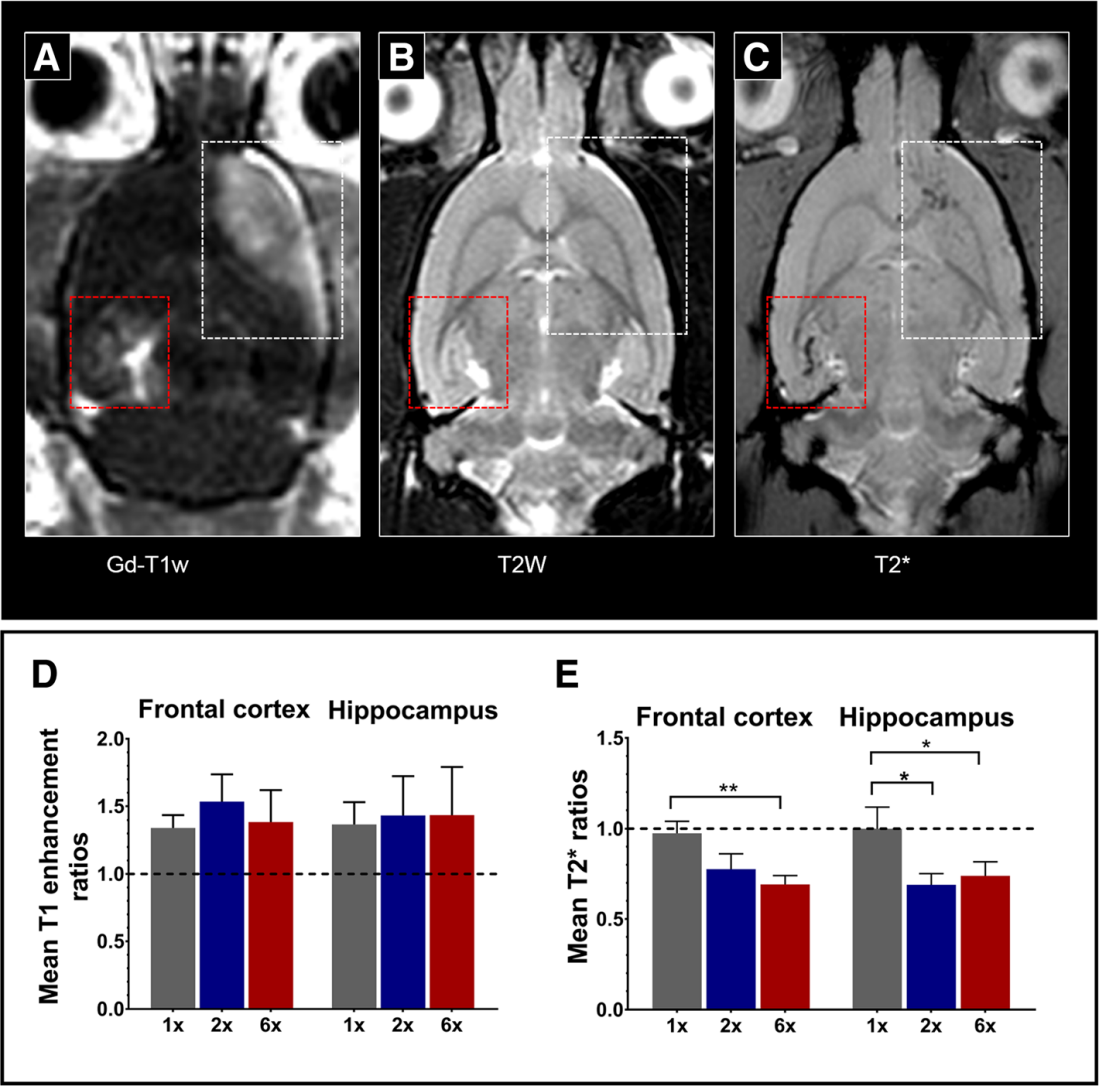
MRI findings in rats treated with pFUS+MB (**a**) T1-weighted scan obtained immediately following the first sonication showing contrast extravasation in the sonicated left frontal cortical (white rectangle) and right hippocampal region (red rectangle), reflecting efficient BBB disruption. **b** T2-weighted scan acquired after two sonications (week 3) showing no significant parenchymal signal abnormalities. **c** T2*-weighted scan obtained after six sonications (week 7) showing multiple hypointense foci in the sonicated regions, likely reflecting a combination of extravasated red blood cells, microhemorrhagic changes, and venous dilatation. **d** Mean ratios of T1 enhancement (sonicated/non-sonicated) in the three groups for one (1×), two (2×), and six (6×) sonications. No statistically significant differences were detected (Kruskal-Wallis test, *p* > 0.05). **e** Mean T2* ratios (sonicated/non-sonicated) in the three groups showing significant incremental decrease in T2* ratios in the sonicated regions with increasing number of sonications (Kruskal-Wallis test, *p* < 0.05, post-hoc Dunn’s test: ** denotes *p* < 0.005 and * denotes *p* < 0.05). Error bars represent standard deviations

In vivo T2*-weighted MRI scans were performed on a Philips 3 T scanner (group A), Bruker 9.4T (Bruker Corp., Billerica, MA) (group B), and Bruker 7T (Bruker Corp., Billerica, MA) (group C) depending on the availability of the resources. For group A animals, axial T2*-weighted images were acquired using a 3-cm-diameter solenoid coil (Philips Research Laboratories, Amsterdam, Netherlands) and fast field echo (FFE) sequence with following parameters: TR/TE = 1301/7 ms, ΔTE = 7 ms, number of echoes = 5, in-plane resolution = 100 × 100 μm^2^, and slice thickness = 500 μm. T2*-weighted images for group B were acquired using a multiple gradient echo (MGE) sequence with TR/TE = 50/3 ms, ΔTE = 3 ms, number of echoes = 10, image resolution = 200 μm^3^ (isotropic) with a 5.0-cm Doty quadrature coil (Doty Scientific, Inc., Columbia, SC). For group C animals, T2*-weighted images were obtained using a 3.8-cm Doty quadrature coil using MGE sequence with TR/TE = 60/2.4 ms, ΔTE = 4.7 ms, number of echoes = 10, image resolution = 200 × 200 μm^2^, and slice thickness = 500 μm (Fig. 2c). The final T2*-weighted images were generated by combining the multiple echo data to enhance the T2* contrast for the evaluation of abnormality.

In four animals from group A, high-resolution ex vivo MRI scans were performed following the imaging sessions, within 1 day after the sonication session. Following perfusion with 4% paraformaldehyde (PFA) in phosphate-buffered saline (PBS), the brains were extracted and immersed in perfluoropolyether fluorinated fluids (PFPE, Galden HS/260, Solvay Solexis, Brussels, Belgium) for scans. The ex vivo scans were performed on a Bruker 7 T scanner with T2-weighted RARE (TR/TE = 2747/15 ms, RARE factor = 4, in-plane resolution = 78 × 78 μm^2^ with 500 μm thickness) and T2*-weighted MGE (TR/TE = 100/5 ms, ΔTE = 5 ms, number of echoes = 14, flip angle = 30°, image resolution = 100 μm^3^ isotropic).

In order to confirm reversal of the BBBO at the time of our earliest PET scan (24 h after sonication), five rats were imaged immediately after sonication (post contrast T1-weighted imaging) as well as 24 h after sonication (pre and post contrast T1-weighted images).

### [18F]DPA-714 radiosynthesis and PET imaging

[18F]DPA-714 was synthesized as previously described [35]. The radiochemical yield was 40–60% (*n* > 20); radiochemical purity was > 99%; molar activity was 33–259 GBq/μmol at the end of synthesis. The concentration used for our experiments was 805 ± 240 MBq/mL (21.75 ± 6.48 mCi/mL) and molar activity during production was 116 ± 64 GBq/μmol (3129 ± 1737 Ci/mmol).

For all PET imaging studies, rats were first anesthetized with 2–2.5% isoflurane–100% oxygen mixture. Anesthesia levels were adjusted to maintain a target respiratory rate of 40–60 breaths/min. Body temperature was maintained by a heating pad.

A preclinical Inveon PET/CT scanner (Siemens Medical Solutions, USA) was used with following imaging parameters: transaxial and axial field of view (FOV) of 10 and 12.7 cm, full width at half maximum spatial resolution at center FOV = 1.4 mm. Two to three rats were scanned per imaging session. [18F]DPA-714 was injected through the tail vein (mean dose = 35.2 MBq (0.951 mCi), mean mass dose at time of injection = 1.46 ± 0.3 nmol/kg) over a period of 30 s as a bolus followed by a quick saline flush (300 μL). Thirty minutes after the injection of the radiotracer, the animal was secured to the scanner bed with its head placed symmetrically within the center FOV. Following CT acquisition for attenuation correction, PET emission scans were acquired in list mode starting at 40 min after injection. The choice for imaging time range is based on our previous experience with this ligand where we found that those frames (40–60 min) reflect “pseudo-equilibrium” status since they had the lowest rate of change in the concentration activity curve (< 5%/h) [36]. The emission sinograms were corrected for scatter, ^18^F-decay, random, and dead time. The resulting histograms were then reconstructed applying Fourier rebinning and 3D ordered subject expectation maximization algorithm (OSEM-3D; 4 OSEM iterations, 18 MAP iterations, matrix: 128 × 128, target resolution: 0.8 mm^2^). The animal was allowed to recover from anesthesia under a heat lamp after scan completion.

### MRI and PET image analysis

Contrast enhancement on T1-weighted images was assessed immediately after the pFUS+MB in the sonicated frontal cortex and hippocampal region compared to the contralateral brain using ImageJ (NIH, Bethesda, USA). Regions of interest (ROIs) were first drawn in the sonicated regions and duplicated on the contralateral side. The ratios of enhancement in the sonicated regions compared to the contralateral brain were calculated using the mean image intensities.

Quantitative T2* maps were created by fitting the signal intensity (S) of each voxel from the MGE images to a mono-exponential decay as a function of TE: *S*_i_ = *S*_0_ exp.(-TE_i_/T2*).

Volumes of interest (VOIs) encompassing sonicated and contralateral regions were drawn to extract the T2* values. The T2* value ratios in the sonicated brain regions compared to the contralateral brain were then calculated from the T2* maps.

Analysis of the reconstructed PET images was done using PMOD 3.7 (PMOD technologies, Ltd., Zurich, Switzerland). First, PET images were co-registered to an MRI template. After co-registration, VOIs were drawn in the sonicated left frontal and right hippocampal regions and mirrored in the corresponding contralateral brain regions, using MR images from the sonicated animals for guidance. Control VOIs were drawn in both cerebellar hemispheres which were not sonicated. Care was taken to avoid including the choroid plexus within the lateral and fourth ventricles (known to have high TSPO expression [37]) and to avoid spillover radioactivity from extracranial structures with high uptake. Averaged percent injected dose/cc (%ID/cc) were derived for these respective VOIs.

### Histological staining and analysis

Rats (*n* = 11) including four from group A, three from group B, and four from group C were euthanized immediately after the last PET imaging session. The animals were perfused with 4% paraformaldehyde fixative (PFA). The brains were then extracted and post-fixed in 4% PFA for 48 h. Fixed tissue blocks were embedded in paraffin wax blocks and sectioned at 3 or 5 μm thickness. Axial brain sections (one section from each animal) including both the sonicated left frontal and right hippocampal regions were stained with Modified Mayer’s Hematoxylin and Eosin-Y (H&E). All slides were counterstained with DAPI (4′,6-diamidino-2-phenylindole) at a concentration of 1 ng/mL to label cell nuclei. Primary antibodies used for IF: chicken anti-glial fibrillary acidic protein (GFAP) 1:200 (AB5541 Millipore Sigma, MA) and rabbit anti-ionized calcium binding adaptor molecule 1 (Iba1) 1:200 (019-19741 Wako Chemicals USA, VA).

Histological evaluation of the microscopy sections was performed at × 20 magnification for all animals in each group. Aperio ScanScope CS equipped with a × 20 air objective (NA = 0.75, Leica Microsystems, Buffalo Grove, IL) was used for microscopy. One section from each brain was used for quantitative analysis. A laser scanning confocal microscope (model 710, Carl Zeiss AG, Oberkochen, Germany) using Plan-Apochromat objectives (× 20 air, NA = 0.8) was used for confocal microscopy. Illumination was provided by argon-ion (Lasos, Jena, Germany), diode and diode-pumped solid-state lasers (Roithner Lasertechnik, Vienna, Austria).

In each rat, ten FOVs were chosen in each of the sonicated regions and ten FOVs were chosen in the exact contralateral brain regions. Thresholds were selected to remove background signal, with the same threshold applied for all regions in the same animal. For each region, fluorescence signal was measured using ImageJ and averaged from all the FOVs within that region. This process was performed sequentially for Iba1 and GFAP staining. Ipsilateral fluorescence signal was then compared to contralateral signal, giving a final value in the form of the ratio of sonicated to non-sonicated regions.

### Statistical analysis

Ratio values of averaged %ID/cc for [18F]DPA-714 PET binding is represented as mean ± standard deviation (SD). Statistical analysis was performed using GraphPad Prism (version.7, GraphPad Software Inc.). For each sonicated region, T1-contrast enhancement ratios, T2* value ratios, and %ID/cc ratios were first calculated. The ratios for staining (sonicated/non-sonicated) were also calculated for Iba1 and GFAP. Kruskal-Wallis non-parametric testing was then used to compare the three groups of animals for significant differences in T1-contrast enhancement, T2* values, and staining intensity. No comparison of PET binding among the three groups was performed due to acquisition of PET scans at different time points after the last sonication. When *p* values were found to be < 0.05, post-hoc Dunn’s multiple comparison tests were performed.

For the animals that underwent repeated imaging after two and six weekly sonications (*n* = 3), %ID/cc ratios were compared using paired *t* test.

The significance threshold for all studies was set at *p* < 0.05.

## Results

### MRI findings

Contrast enhancement on T1-weighted MRI immediately following pFUS+MB was identified in the sonicated regions for all three groups (Fig. 2a, Additional file 1: Figure S1). In five animals scanned 24 h after sonication, we did not identify any contrast enhancement confirming reversal of the BBBO at the time of our earliest PET scan (Additional file 1: Figure S1). Even though all animals showed definite enhancement in the ipsilateral sonicated brain compared to the contralateral side immediately after sonication, the ratios of T1-enhancement (sonicated/non-sonicated) were not significantly different between the groups in either region (*p* > 0.05) (Fig. 2d). The ratios of T2* values (sonicated/non-sonicated) were significantly different between the three groups in the left frontal region (*p* < 0.0001) with post-hoc analysis showing significantly lower ratios in group C (6×) compared to group A (1×) (*p* = 0.0023). The ratios of T2* values were significantly different between groups in the right hippocampal region (*p* = 0.0018) with post-hoc analysis showing significantly lower ratios in group B (2×) compared to group A (1×) (*p* = 0.02) and in group C (6×) compared to group A (*p* = 0.039) (Fig. 2e).

### PET-imaging results

**Fig. 3:**
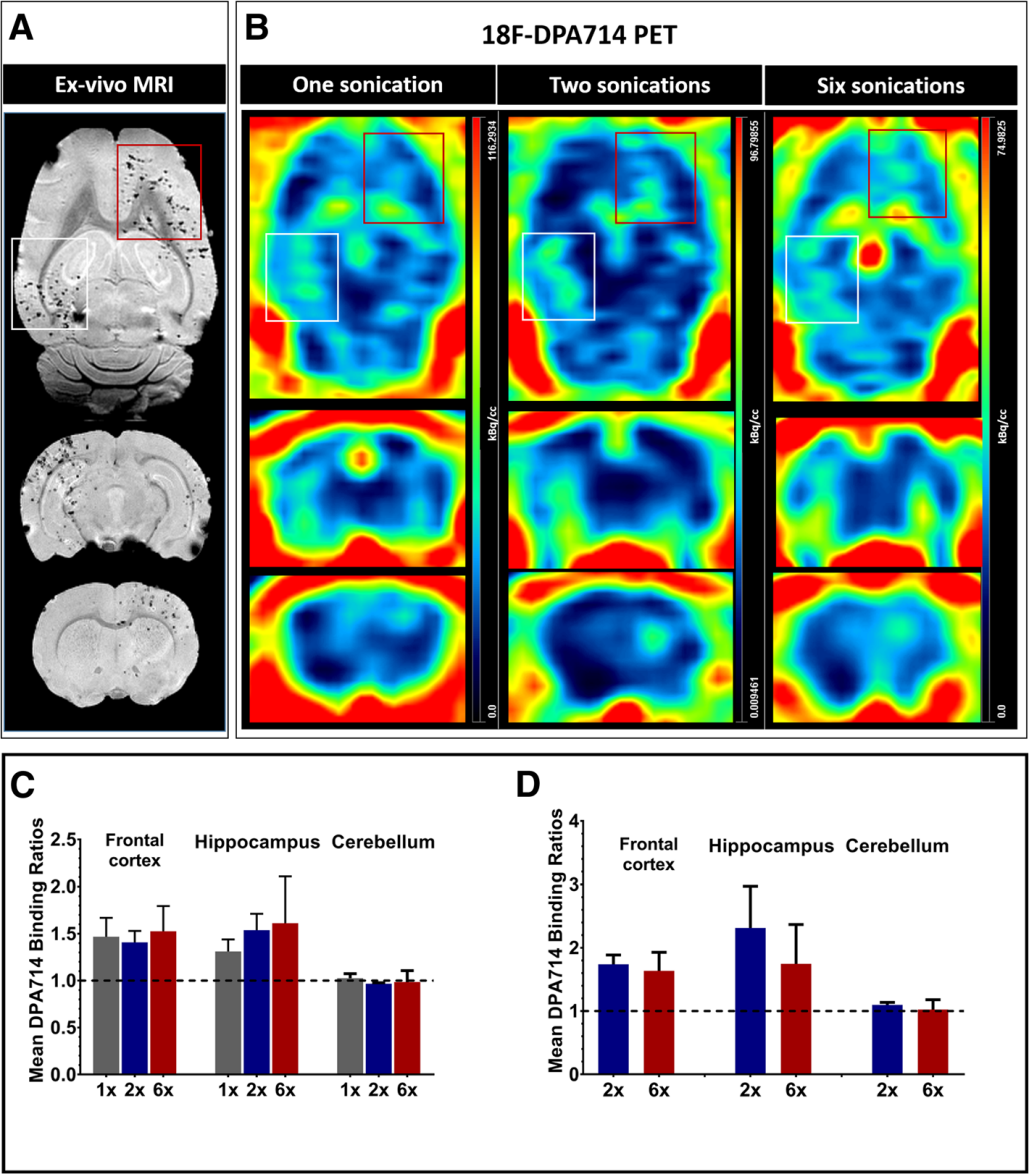
PET imaging findings in rats treated with pFUS+MB (**a**) Ex vivo T2*-weighted MR images showing multiple hypointense foci in the sonicated frontal region (red box) and the right hippocampal region (white box) suggestive of extravasated red blood cells, microhemorrhagic changes and hemosiderin within phagocytic cells. **b** Three examples of [18F]DPA-714 PET scans showing increased binding in the sonicated regions after one, two, and six sonication sessions respectively. **c** Ratios of [18F]DPA-714 binding (sonicated/non-sonicated mean %ID/cc values) in both sonicated regions following one (1×), two (2×), and six (6×) sonications. No increased [18F]DPA-714 seen in the cerebellar hemispheres which were not sonicated. **d** Longitudinal mean ratios of [18F]DPA-714 binding in a subset of animals after two (2×) and six (6×) sonications. No statistically significant differences were detected in the sonicated regions or in the cerebellum (control region) (Paired t-test, *p* > 0.05). Error bars represent standard deviations

In all groups, [18F]DPA-714 PET imaging showed mean %ID/cc ratios (sonicated/non-sonicated) that are > 1 in the frontal cortical and hippocampal regions (Fig. 3, Tables 1 and 2). [18F]DPA-714 binding between the two cerebellar hemispheres was very similar for all three groups (ratio range of 0.98–1.03) (Table 1, Fig. 3c).

**Table 1:** Mean ratio of [18F]DPA-714 binding (sonicated/non-sonicated) in the frontal cortex and hippocampus in all three groups of animals. The cerebellum was not sonicated and is used as control

Region of interest	Mean ratio of ipsilateral/contralateral		
	1 sonication *N* = 6	2 sonications *N* = 8	6 sonications *N* = 5
Frontal cortex	1.47 ± 0.2	1.53 ± 0.2	1.53 ± 0.3
Hippocampus	1.31 ± 0 .1	1.83 ± 0.5	1.61 ± 0.5
Cerebellum	1.03 ± 0.04	1.02 ± 0.07	0.98 ± 0.12

**Table 2:** Mean ratio of [18F]DPA-714 binding (sonicated/non-sonicated) in the frontal cortex and hippocampus in animals that were followed longitudinally after two and six sonications. The cerebellum was not sonicated and is used as control

Region of interest	Mean ratio of ipsilateral/contralateral	
	2 sonications	6 sonications
Frontal cortex	1.74 ± 0.1	1.64 ± 0.3
Hippocampus	2.31 ± 0.7	1.74 ± 0.6
Cerebellum	1.10 ± 0.04	1.02 ± 0.1

In a longitudinal analysis of three rats imaged after the second and then after the sixth sonication, the mean %ID/cc ratios were not significantly different in either region nor in the cerebellum (*p* > 0.05) (Fig. 3d).

### Histology findings in pFUS+MB-treated rats

**Fig. 4:**
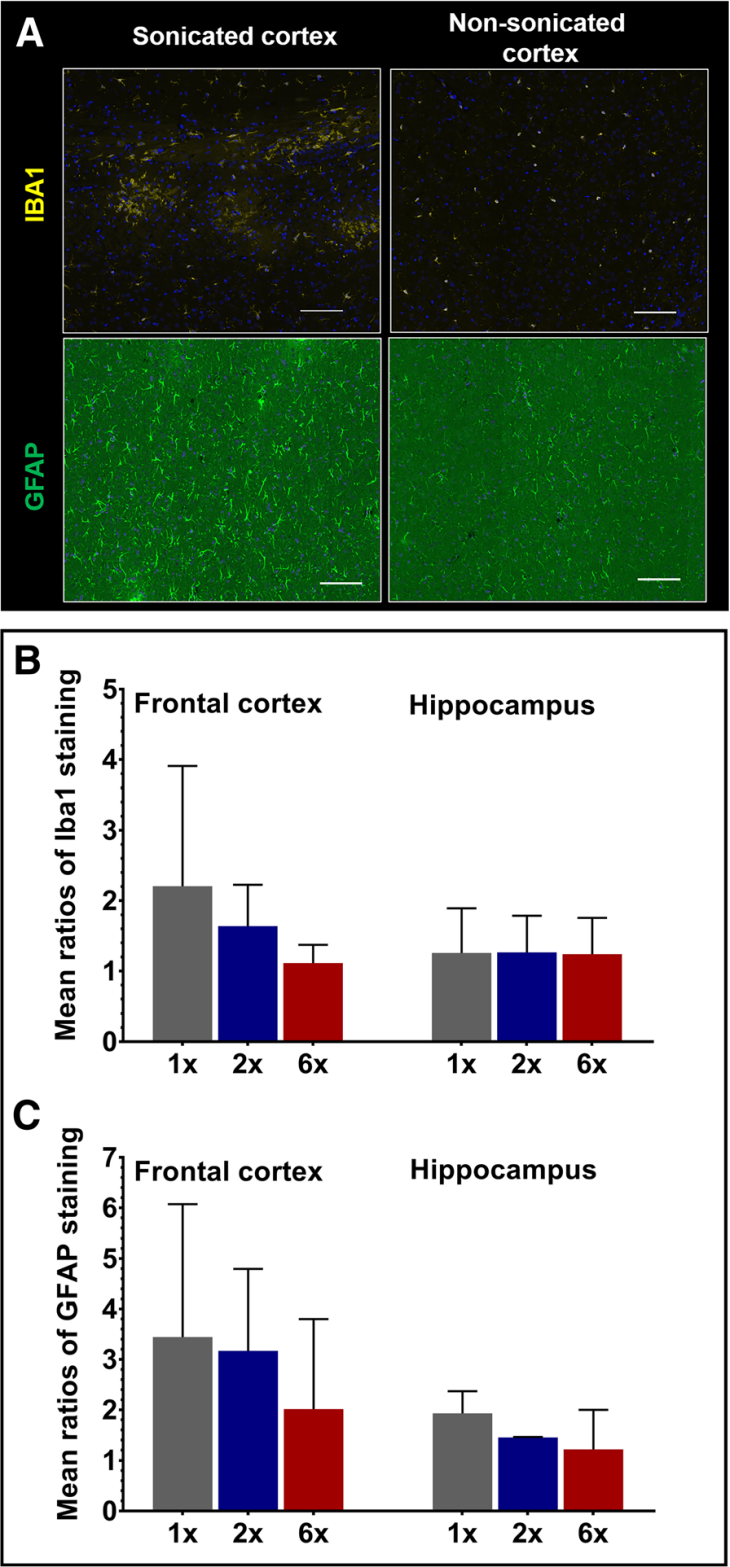
Histopathologic correlates in pFUS+MB treated rats. **a** Iba1 and GFAP staining from sonicated and contralateral non-sonicated brain (frontal cortex) showing patchy foci of increased Iba1 staining as well as more diffuse increase in GFAP staining. **b** Mean Iba1 and **c** mean GFAP fluorescence signal ratios (sonicated/non-sonicated) in the frontal and hippocampal regions respectively. Error bars represent standard deviations

Increased GFAP and Iba1 staining related to pFUS+MB was noted in the sonicated regions compared to the non-sonicated (contralateral) brain in all three groups with representative examples shown in Fig. 4a. The averaged ratios (sonicated/non-sonicated fluorescent signal) were >1 in both regions; however, the changes were more impressive in the sonicated frontal cortex than in the sonicated hippocampus (compared to respective non-sonicated brains) (Fig. 4b, c). Those differences however did not reach significance in either location (*p* > 0.05).

## Discussion

MRIgFUS with MB is a proposed approach to open the BBB for purposes of drug delivery in specific targeted brain regions [8–12, 38, 39]. The mechanism of BBBO by pFUS+MB is likely due to acoustic radiation pressure generated by ultrasound along with acoustic stable or inertial cavitation effects from the intravascular MB oscillations. Secondary stretching of the endothelial cells coupled with induced expression of cytokines, chemokines, and trophic factors (CCTF) from the various cellular components of the NVU and alterations in tight junction integrity eventually result in BBBO [14, 40, 41]. pFUS+MB-induced transient BBBO has already been used for neurotherapeutics’ delivery in experimental models of disease [42–44] and has been proposed to increase beta-amyloid (Aβ) clearance from the brains of AD mouse models [45–47]. More recently, serial pFUS+MB has been used in various clinical trials to open the BBB (clinicaltrials.gov: NCT02986932, NCT03119961, NCT03347084), mainly as a method to decrease Aβ deposition in patients with AD [20], and in patients with CNS malignancy [21]. Despite the purported benefits of pFUS+MB, side effects of this technique, such as potential neuroinflammatory sequelae, have received little attention.

Recent studies have suggested that pFUS+MB can induce a sterile inflammatory response (SIR) as evidenced by increased proteomic and transcriptomic expression of proinflammatory CCTF [14, 24, 33]. In our study, we were able to visualize those neuroinflammatory changes using noninvasive [18F]DPA-714 PET imaging. Our results were not confounded by leakage across a disrupted BBB since by the time we imaged the animals (24 h following first sonication and 1–2 weeks after the second and sixth sonications), the acute BBB disruption by pFUS would have completely resolved [48, 49]. In fact, we confirmed reversal of BBBO in a separate group of five animals by documenting lack of contrast leakage on MRI in the sonicated regions 24 h after sonication (Additional file 1: Figure S1). Our PET imaging findings were further supported by increased Iba1 and GFAP immunofluorescent staining in the sonicated areas consistent with microglial activation and astrocytosis. The increased [18F]DPA-714 binding in the sonication sites was observed as early as 24 h after one sonication (Fig. 3a, b) as well as for days to weeks after two and six weekly pFUS+MB exposures (Fig. 3c). We did not compare binding between the three groups due to differences in time lapses between PET scanning and the last sonication. However, in the longitudinal cohort of animals that was imaged within a comparable time frame after the second and sixth sonications, we did not see significant differences in [18F]DPA-714 binding (Fig. 3d, Table 2) suggesting no PET-detectable evidence of cumulative inflammatory effect. Our findings support the notion of an SIR that persists for at least 2 weeks after the reversal of BBB disruption, although we have no PET evidence of additive effect of multiple sonications based on a small subset of animals imaged longitudinally.

Using T1-weighted imaging, we confirmed successful BBBO immediately post pFUS+MB (Fig. 2a). The degree of enhancement seen immediately post sonication did not significantly change between groups (Fig. 2d) which is not surprising since BBBO is expected to have reversed within the time interval between the sessions [14, 49]. This was confirmed by lack of contrast enhancement in a separate group of animals imaged with MRI 24 h after sonication. There was however an incremental further decrease in T2* values after two and six sonications reaching statistical significance in both sonicated regions when compared to one sonication (Fig. 2e). As previously described, those findings reflect microhemorrhagic changes related to vascular injury and microglia and macrophages (metallophagocytic cells) phagocytosing the red blood cells in the parenchyma. Secondary iron deposition (T2* signal) is detected and increases as a function of the number of sonication sessions [14, 24]. Some of the hypointense voxels also reflect slow blood flow in dilated vessels as previously demonstrated [24]. Of note, hypointense voxels on 3 T T2*-weighted images were recently reported in two out of five AD patients who received pFUS+MB to open the BBB [20].

There have been multiple discussions in the literature related to the comparability of pFUS+MB parameters/dose between the various preclinical studies and their equivalency to human applications. The picture however remains complex with multiple factors affecting the end-result of efficient BBB disruption [14, 18, 24, 33, 50, 51]. In practical terms, the magnitude of BBBO following pFUS+MB is dependent upon multiple parameters including the MB type (size and sonographic characteristics), infusion rate (bolus vs. slow infusion), dose, and initial concentration of injected MB as well as other experimental conditions including oxygenation levels, PNP, and PCD parameters [18, 33, 50–53]. To add to the complexity, there are several approaches for PCD feedback used to limit MB cavitation [54–57], although it is not clear which is the optimal method for controlling PNP changes that would limit parenchymal injury. In our study, we used 100% oxygenation which significantly decreases the half-life of MB in the vasculature (0.72 min compared to 1.43 min at 21% oxygenation) [58–60]. We also used PCD feedback of PNP in order to limit the magnitude of ultraharmonics at 1.5*fo* and 2.5*fo* to less than 3.5 and minimize parenchymal injury by limiting the amount of stable cavitation from intravascular MB [55]. We used Optison at 100 μL (5–8 × 10^7^ MB/rat) independent of weight, infused slowly over 1 min with sonication delayed by 30 s allowing for the MB concentration in the vasculature to reach near steady-state [33]. As the animals increased in age and weight, the effective MB concentration administered to groups B and C decreased as a consequence of increasing weight. Moreover, a recent study has shown that repeated exposures to Optison will significantly shorten the intravascular half-life of the MB [61], which would further decrease the effective dose of MB used in group B and C rats compared to group A rats.

Although it is difficult to compare our procedural parameters to those used in humans [20], the MB dose (total #MB) used in the current study is within the range of preclinical experimental protocol parameters reported in the literature to cause BBBO following pFUS with or without PCD feedback [24, 54, 62, 63]. Most relevant to many of the clinical trials targeting AD-afflicted subjects, our experimental pFUS parameters are comparable to those used in animal studies showing the effectiveness of FUS in clearance of Aβ plaques in mouse models [45–47, 64, 65]. Theoretically, if the clinical protocols using lower concentrations of MB do not achieve a similar level of BBBO as those in the preclinical studies, the effectiveness and human translatability of the approach would be in jeopardy. Limitations associated with human TSPO imaging such as the known polymorphism of the TSPO gene (rs6971) resulting in different binding affinities, should be taken into account; quantification however is simplified considering the focal nature of pFUS+MB allowing the use of contralateral non-sonicated brain as reference region without the extra hassle of arterial blood sampling [26].

As we have previously mentioned [33], the main questions regarding the utility of pFUS in human clinical trials can be summarized as follows: (1) What pFUS parameters are needed to induce the necessary BBB disruption for adequate parenchymal delivery of neurotherapeutics or stimulation of the required immune response? (2) What risks would the combination of those sonication parameters impose as far as inducing a neuroinflammatory reaction with potential secondary tissue damage? Using the current method with parameters that are comparable to those used in most preclinical studies [24], we have shown an SIR in animals undergoing MRI-guided pFUS+MB opening of the BBB in vivo, using PET imaging, as early as 24 h after single sonication. Multiple sonications resulted in worsening of the MRI findings especially hemosiderin deposition. However, there was no statistically significant increase in the associated SIR, neither by imaging nor by histology. This could suggest resolution of the inflammatory changes in-between sessions.

## Conclusion

Considering the great clinical potential of pFUS+MB, our findings warrant a deeper exploration of the optimal experimental parameters in human clinical trials needed to induce a therapeutically useful degree of BBBO but without the potentially harmful neuroinflammatory effects. [18F]DPA-714 PET along with MRI could be used to determine those optimal pFUS+MB parameters which would minimize the negative effects of the intervention while achieving the necessary BBBO.

## Additional file

Additional file 1: **Figure S1.** MRI scans obtained immediately after sonication show contrast leakage in the left frontal region (red box) and the right hippocampal region (white box). Repeat imaging after 24 h shows resolution of contrast leakage consistent with reversal of BBB opening. (TIF 784 kb)

## Data Availability

All reported data can be made available to qualified investigators upon request.

## Abbreviations

%ID/cc: Percent injected dose/cc
AD: Alzheimer’s disease
BBB: Blood-brain barrier
BBBO: Blood-brain barrier opening
CCTF: Cytokines, chemokines, and trophic factors
DAMP: Damage-associated molecular pattern
FFE: Fast field echo
FFT: Fast Fourier transform
FOV: Field of view
GFAP: Glial fibrillary acidic protein
Iba1: Ionized calcium binding adaptor molecule 1
MB: Microbubbles
MGE: Multiple gradient echo
MRI: Magnetic resonance imaging
NVU: Neurovascular unit
OSEM: Ordered subject expectation maximization algorithm
PBS: Phosphate-buffered saline
PCD: Passive cavitation detection feedback
PFA: Paraformaldehyde
PFPE: Perfluoropolyether fluorinated fluids
pFUS+MB: Pulsed focused ultrasound + microbubbles
PNP: Peak negative pressure
PRF: Pulse repetition frequency
ROI: Region of interest
SIR: Sterile inflammatory response
TR/TE: Repetition time/echo time
TSPO: Translocator protein
VOI: Volume of interest
