# Supplementary figures and images for: In vivo imaging of sterile microglial activation in rat brain after disrupting the blood-brain barrier with pulsed focused ultrasound: [18F]DPA-714 PET study
Source: J Neuroinflammation. 2019 Jul 25;16:155. doi: 10.1186/s12974-019-1543-z (PMC6657093; doi:10.1186/s12974-019-1543-z)

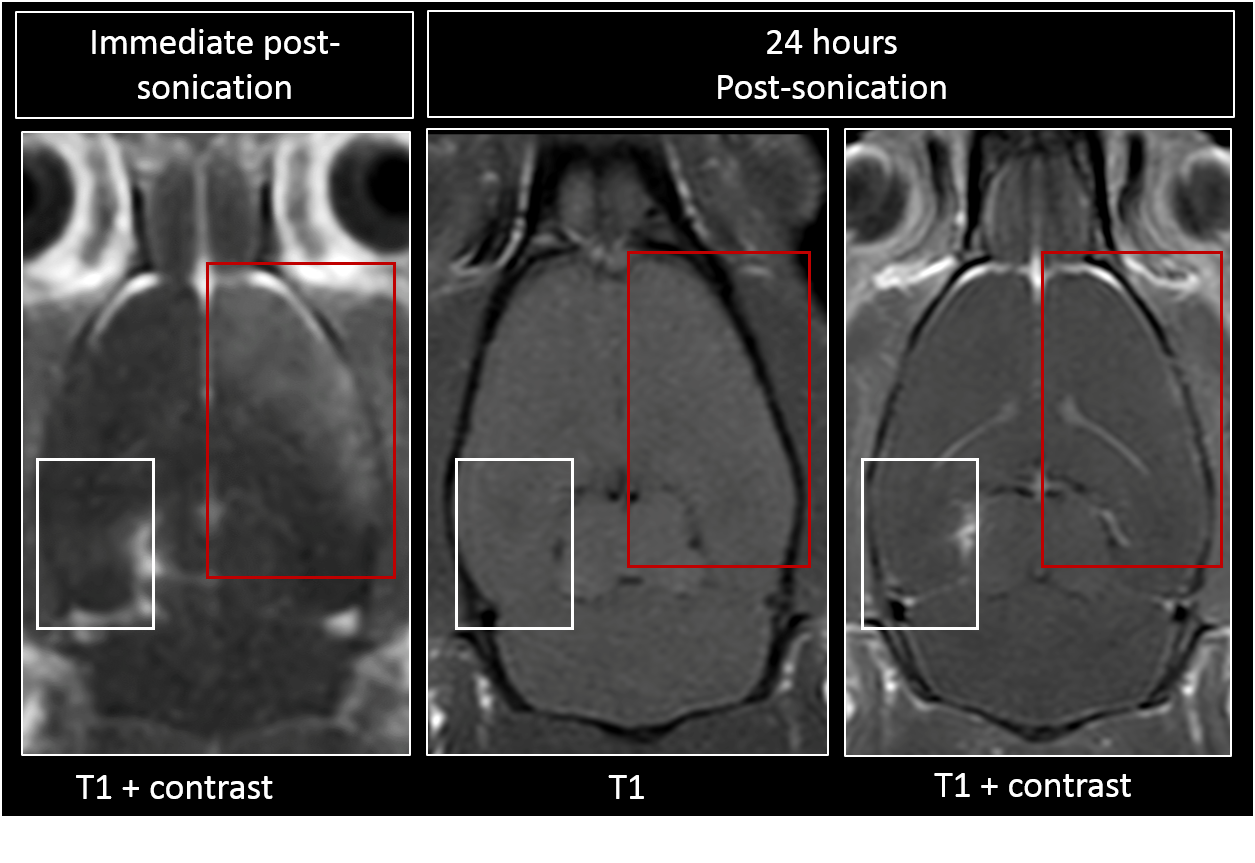

Supplement: Supplementary file 1 — Figure S1. MRI scans obtained immediately after sonication show contrast leakage in the left frontal region (red box) and the right hippocampal region (white box). Repeat imaging after 24 h shows resolution of contrast leakage consistent with reversal of BBB opening. (TIF 784 kb) [file 12974_2019_1543_MOESM1_ESM.tif]
